# Supplementary material for: Fast-decaying plant litter enhances soil carbon in temperate forests but not through microbial physiological traits
Source: Nat Commun. 2022 Mar 9;13:1229. doi: 10.1038/s41467-022-28715-9 (PMC8907208; doi:10.1038/s41467-022-28715-9)
Supplement: Supplementary file 1 — Supplementary Information [file 41467_2022_28715_MOESM1_ESM.pdf]

Supplementary information for

Fast-decaying plant litter enhances soil carbon in temperate forests, but not through microbial physiological traits

Matthew E. Craig[1,2,*], Kevin M. Geyer[3,4], Katilyn V. Beidler[1], Eddie R. Brzostek[5], Serita D. Frey[3], A. Stuart Grandy[3], Chao Liang[6], Richard P. Phillips[1]

[1]Department of Biology, Indiana University, Bloomington, IN, USA
[2]Environmental Sciences Division and Climate Change Science Institute, Oak Ridge National Laboratory, Oak Ridge, TN, USA
[3]Department of Natural Resources and the Environment, University of New Hampshire, Durham, NH, USA
[4]Department of Biology, Young Harris College, Young Harris, GA, USA
[5]Department of Biology, West Virginia University, Morgantown, WV, USA
[6]Key Laboratory of Forest Ecology and Management, Institute of Applied Ecology, Chinese Academy of Sciences, Shenyang, China

[*]*Correspondence:* craigme@ornl.gov

This file contains:
Supplementary tables 1-6
Supplementary figures 1-10

Table S1. Indicators of leaf litter quality (mean, SE, *n* = 3), estimates and standard error for decomposition parameters (s, k_1, and k_2) derived from the double exponential model— $C\ remaining = se^{-k_1 t} + (1-s)e^{-k_2 t}$; where t is time in days—and average (± standard error, *n* = 4) percent carbon (C) loss after 185 days of decomposition for the sixteen species used in this study. Litter quality indicators include the percentage nitrogen (N), soluble fraction, and acid unhydrolyzable residue (AUR) fraction by mass, lignocellulose index (LCI) and the AUR:N ratio.

Species	C:N	% N	% Soluble	% AUR	LCI	AUR:N	s	k_1	k_2	% C loss
Acer rubrum	89.7 (4.6)	0.52 (0.03)	61.7 (0.2)	7.0 (2.4)	0.19 (0.06)	13.1 (4.1)	0.31 (0.01)	0.15 (0.01)	0.0036 (0.0002)	62.2 (0.7)
Acer saccharum	49.6 (2.6)	0.89 (0.05)	50.4 (0.7)	12.4 (0.2)	0.27 (0.00)	14.1 (0.7)	0.27 (0.02)	0.14 (0.03)	0.0038 (0.0004)	60.8 (3.8)
Asimina triloba	21.7 (0.4)	2.09 (0.04)	39.7 (0.2)	15.6 (0.4)	0.26 (0.01)	7.4 (0.3)	0.27 (0.01)	0.11 (0.01)	0.0006 (0.0001)	34.1 (0.9)
Carya cordiformis	42.7 (1.8)	1.05 (0.05)	32.1 (1.4)	20.7 (0.8)	0.31 (0.01)	19.9 (0.9)	0.27 (0.01)	0.09 (0.01)	0.0012 (0.0001)	39.9 (0.3)
Carya glabra	50.2 (0.8)	0.89 (0.02)	29.1 (0.6)	22.7 (0.6)	0.32 (0.01)	25.5 (1.2)	0.27 (0.01)	0.07 (0.00)	0.0010 (0.0001)	38.4 (0.7)
Carya ovata	47.2 (3.5)	0.95 (0.08)	34.1 (1.4)	20.6 (0.4)	0.32 (0.00)	22.1 (2.0)	0.32 (0.03)	0.07 (0.01)	0.0015 (0.0004)	48.0 (4.1)
Fagus grandifolia	49.3 (1.0)	0.92 (0.02)	26.6 (0.4)	22.9 (0.2)	0.25 (0.01)	24.9 (0.6)	0.22 (0.01)	0.05 (0.01)	0.0017 (0.0001)	42.5 (1.2)
Fraxinus americana	39.8 (1.1)	1.04 (0.08)	41.7 (0.8)	14.5 (0.9)	0.32 (0.00)	14.1 (1.1)	0.29 (0.01)	0.15 (0.01)	0.0014 (0.0002)	43.4 (1.7)
Liriodendron tulipifera	57.6 (2.1)	0.78 (0.03)	52.0 (0.7)	13.7 (0.4)	0.29 (0.00)	17.7 (1.1)	0.23 (0.01)	0.16 (0.01)	0.0007 (0.0001)	31.6 (0.6)
Nyssa sylvatica	64.2 (1.2)	0.71 (0.02)	54.4 (1.1)	9.9 (0.2)	0.22 (0.01)	14.1 (0.3)	0.23 (0.01)	0.13 (0.01)	0.0033 (0.0001)	55.2 (0.7)
Quercus alba	57.7 (0.7)	0.82 (0.01)	50.3 (1.4)	15.0 (0.4)	0.31 (0.00)	18.2 (0.4)	0.36 (0.08)	0.03 (0.01)	0.0011 (0.0008)	47.4 (3.2)
Quercus prinus	74.9 (1.2)	0.63 (0.01)	48.0 (1.0)	17.3 (0.2)	0.35 (0.01)	27.5 (0.7)	0.22 (0.01)	0.06 (0.01)	0.0017 (0.0001)	42.5 (1.1)
Quercus rubra	71.0 (4.8)	0.69 (0.05)	35.9 (1.2)	24.5 (0.3)	0.39 (0.01)	36.1 (2.7)	0.23 (0.01)	0.07 (0.01)	0.0015 (0.0001)	40.3 (1.8)
Quercus velutina	61.6 (4.8)	0.79 (0.06)	40.4 (0.7)	21.8 (0.4)	0.37 (0.00)	28.1 (2.0)	0.22 (0.01)	0.06 (0.01)	0.0012 (0.0001)	36.9 (1.4)
Sassafras albidum	57.7 (3.6)	0.83 (0.04)	54.0 (0.9)	15.4 (0.7)	0.34 (0.02)	18.6 (1.9)	0.16 (0.01)	0.16 (0.02)	0.0011 (0.0001)	30.2 (0.7)
Tilia americana	27.5 (0.8)	1.69 (0.06)	42.7 (1.2)	23.9 (2.2)	0.42 (0.03)	14.2 (1.6)	0.22 (0.01)	0.11 (0.01)	0.0010 (0.0001)	33.9 (1.5)

Table S2 Average values (± 1 SE; *n* = 4) for microbial growth rate (MGR; µg C g soil^{-1} d^{-1}), carbon use efficiency (CUE), and microbial turnover rate (MTR; d^{-1}) for soils exposed to different leaf litter species at an early (15 days) and intermediate (intermed.;100 days) stage of decomposition.

	MGR		CUE		MTR	
Species	Early	Intermed.	Early	Intermed.	Early	Intermed.
Acer rubrum	195.7 (5.0)	46.8 (4.1)	0.53 (0.04)	0.17 (0.02)	0.074 (0.002)	0.047 (0.004)
Acer saccharum	182.0 (15.4)	75.4 (5.4)	0.50 (0.07)	0.29 (0.02)	0.058 (0.005)	0.067 (0.005)
Asimina triloba	79.9 (15.1)	19.4 (4.6)	0.33 (0.05)	0.14 (0.03)	0.057 (0.011)	0.033 (0.008)
Carya cordiformis	86.9 (4.3)	25.1 (2.3)	0.35 (0.04)	0.15 (0.01)	0.048 (0.002)	0.038 (0.004)
Carya glabra	70.6 (7.6)	13.7 (1.9)	0.30 (0.03)	0.11 (0.04)	0.054 (0.006)	0.029 (0.004)
Carya ovata	110.9 (8.4)	27.1 (2.0)	0.33 (0.03)	0.14 (0.01)	0.072 (0.005)	0.040 (0.003)
Fagus grandifolia	124.1 (5.0)	37.8 (3.1)	0.33 (0.05)	0.19 (0.02)	0.075 (0.003)	0.040 (0.003)
Fraxinus americana	101.2 (5.4)	25.9 (5.0)	0.37 (0.08)	0.14 (0.03)	0.070 (0.004)	0.045 (0.009)
Liriodendron tulipifera	104.1 (7.7)	45.1 (3.8)	0.35 (0.05)	0.25 (0.02)	0.052 (0.004)	0.040 (0.003)
Nyssa sylvatica	112.5 (7.3)	70.4 (7.5)	0.37 (0.03)	0.23 (0.01)	0.052 (0.003)	0.050 (0.005)
Quercus alba	187.2 (14)	48.9 (5.2)	0.39 (0.03)	0.17 (0.02)	0.071 (0.005)	0.042 (0.004)
Quercus prinus	121.2 (7.1)	34.5 (8.7)	0.49 (0.03)	0.19 (0.04)	0.072 (0.004)	0.035 (0.009)
Quercus rubra	87.2 (3.8)	22.5 (4.0)	0.37 (0.07)	0.13 (0.02)	0.058 (0.003)	0.034 (0.006)
Quercus velutina	142.0 (26.8)	45.6 (6.0)	0.38 (0.08)	0.23 (0.03)	0.085 (0.016)	0.048 (0.006)
Sassafras albidum	65.9 (9.3)	22.8 (2.4)	0.27 (0.03)	0.21 (0.03)	0.050 (0.007)	0.043 (0.005)
Tilia americana	87.9 (15.2)	33.8 (5.3)	0.37 (0.09)	0.21 (0.03)	0.065 (0.011)	0.036 (0.006)
Control	10.6 (0.6)	2.3 (0.2)	0.26 (0.03)	0.06 (0.01)	0.024 (0.001)	0.015 (0.002)

Table S3 Mean values (± 1 SD) for soil biogeochemical variables at each site (n = 9) – Wabikon Lake Forest (WLF), Harvard Forest (HF), Lilly-Dickey Woods (LDW), Smithsonian Conservation Biology Institute (SCBI), Smithsonian Environmental Research Center (SERC), and Tyson Research Center (TRC) – and overall (n = 54). Significant ($p < 0.05$) positive [+] and negative [-] Pearson's correlations with ectomycorrhizal dominance are also indicated. Variables include dissolved organic carbon (DOC) and the acid unhydrolyzable residue-to-nitrogen ratio (AUR:N).

Site	Soil C:N	pH	N mineralization (mg N g soil^{-1} d^{-1})	DOC (µg g soil^{-1})	Leaf litter AUR:N	Fine root biomass
WLF	14 (2)	4.8 (0.5)	-1.1 (3.3) [-]	92 (124)	22 (1) [+]	19.6 (4.8)
HF	21 (4) [+]	3.7 (0.4)	0.7 (0.7)	151 (65) [+]	24 (4)	8.5 (3.2) [-]
LDW	16 (4) [+]	4.4 (1.0) [-]	0.2 (1.1) [-]	52 (30) [+]	22 (3)	9.8 (6.0) [+]
SCBI	14 (3) [+]	5.2 (1.0) [-]	0.6 (0.3)	35 (8) [+]	23 (4) [+]	9.8 (4.1)
SRC	13 (1) [+]	4.1 (0.8)	0.3 (0.3) [-]	151 (146)	29 (4)	11.0 (3.6)
TRC	14 (2)	5.6 (0.6)	0.7 (0.6)	25 (7)	22 (5)	5.5 (2.7)
Overall	15 (4) [+]	4.6 (1.0) [-]	0.2 (1.5) [-]	84 (95)	24 (4) [+]	11 (6)

Table S4 Mean values (± 1 SD; n = 9) for microbial biomass carbon (MBC), microbial growth rate (MGR), microbial carbon use efficiency (CUE), microbial turnover rate (MTR), and microbial necromass concentrations at the study sites. Necromass is reported on a "per mass soil" and "per mass soil C" basis and fungal-to-bacterial ratios (F:B) are also shown. See Table S3 for additional abbreviations.

Site	MGR (µg C g soil^{-1} d^{-1})	CUE	MTR (yr^{-1})	MBC (µg C g soil^{-1})	Necromass (mg C g soil^{-1})	Necromass (% of soil C)	Necromass F:B
WLF	13.0 (12.6)	0.15 (0.05)	2.9 (1.8)	1568 (186)	39.2 (11.9)	53.2 (17)	6.9 (1.8)
HF	13.0 (7.1)	0.18 (0.05)	2.5 (1.0)	1810 (178)	25.9 (12.2)	22.6 (13.6) [-]	5.1 (2)
LDW	10.6 (7.2) [+]	0.20 (0.05) [+]	3.6 (1.0)	1019 (139)	17.4 (5.9)	44.1 (11.1) [-]	7.3 (0.8)
SCBI	7.6 (2.1)	0.17 (0.03)	3.3 (1.4) [+]	902 (71) [-]	15.0 (4.3)	43.9 (8.5)	6.6 (1.5) [+]
SRC	3.4 (1.8)	0.14 (0.07) [-]	1.9 (0.8)	636 (50)	13.7 (3)	52.1 (10.1)	3.0 (0.7)
TRC	4.7 (1.8)	0.14 (0.03) [+]	2.2 (0.8)	780 (40)	15.5 (2.7)	46.7 (7.5)	5.1 (1.4)
Overall	8.7 (7.5)	0.16 (0.05)	2.7 (1.3) [+]	1119 (559)	21 (12)	44 (15)	5.7 (2.0)

Table S5 Climatic and mean edaphic properties (± 1 SD; *n* = 9) at the study sites including mean annual temperature (MAT) and precipitation (MAP), oxalate-extractable iron (Fe_{ox}) and aluminum (Al_{ox}), total carbon (Tot-C) and mineral-associated organic carbon (MA-SOC) concentrations, and the proportion of SOC stored in MA-SOC (MA-SOC/Tot-C). See Table S3 for site abbreviations.

Site	MAT (°C)	MAP (mm yr^{-1})	Sand (%)	Silt (%)	Clay (%)	Fe_{ox}[a]	Al_{ox}[a]	Tot-C[a]	MA-SOC[a]	MA-SOC / Tot-C
WLF	4.2	805	37 (22)	56 (21)	7 (2)	2.8 (1.5)	0.9 (1.1)	81 (32)	18 (5)	0.26 (0.14)
HF	9	1050	63 (4)	29 (4)	8 (2)	5.1 (1.3)	2.4 (0.9)	131 (63)	25 (10)	0.24 (0.12)
LDW	11.6	1203	15 (10)	76 (10)	9 (1)	1.6 (0.6)	1.2 (0.8)	43 (22)	14 (5)	0.37 (0.11)
SCBI	12.9	1001	26 (6)	60 (6)	14 (2)	2.6 (0.8)	1.8 (1.1)	34 (7)	16 (4)	0.48 (0.11)
SRC	13.2	1068	50 (9)	35 (9)	15 (4)	2.5 (1.0)	1.6 (0.9)	26 (4)	13 (2)	0.51 (0.10)
TRC	13.5	957	9 (6)	82 (5)	9 (2)	1.7 (0.4)	1.4 (0.8)	34 (9)	14 (3)	0.41 (0.07)

Climatic data obtained from Anderson-Teixeira et al. (2015)

[a]mg g $soil^{-1}$

Table S6 Percentage of total basal area for common (> 0.5 % basal area) arbuscular mycorrhizal trees (AM species) and ectomycorrhizal trees (ECM species) summed across study plots at each site. See Table S3 for abbreviations.

		% Basal area					
	Species	HF	LDW	SCBI	SERC	TRC	WLF
**AM Species**	*Liriondendron tilipifera*		8.4	54.5	19.7		
	Acer saccharum	14.2	14.4			0.7	40.0
	Fraxinus americana	21.4	7.5	1.9		14.0	5.5
	Acer rubrum	11.2	4.9		3.2	1.0	
	Liquidambar styraciflua				17.7		
	Ulmus rubra			0.6		7.2	
	Nyssa sylvatica			1.1	6.5		
	Sassafras albidum		1.9			4.5	
	Juglans nigra					4.7	
	Cornus florida					3.3	
	Celtis occidentalis					2.6	
	Lindera Benzoin				0.5	1.7	
	Fraxinus nigra						2.0
	Fraxinus caroliniana					1.6	
	Asimina triloba					1.3	
	Ailanthus altissima					1.1	
	Prunus serotina	0.5					
	Cercis canadensis					0.5	
**ECM Species**	*Quercus rubra*	32.7	15.8	8.6	2.5	9.3	
	Quercus alba		3.3	9.4	14.4	28.2	
	Quercus prinus		35.1				
	Quercus velutina		3.2	8.1	6.0	9.1	
	Fagus grandifolia		1.0	2.6	20.8		
	Tilia americana						20.3
	Carya glabra		2.6	7.6	4.1	2.9	
	Betula alleghaniensis						11.8
	Populus tremuloides						10.6
	Pinus strobus	7.6		1.1			
	Tsuga species	7.6					
	Carya tomentosa			1.2		5.1	
	Populus grandidentata						6.3
	Carya ovalis		1.7	0.7		0.9	
	Carya alba				2.6		
	Ostrya virginiana						2.5
	Betula lenta	2.1					
	Carpinus caroliniana				1.1		
	Betula alba	0.9					
	Pinus pungens			0.9			
	Picea glauca						0.7
	Betula papyrifera	0.6					

Supplementary Figures

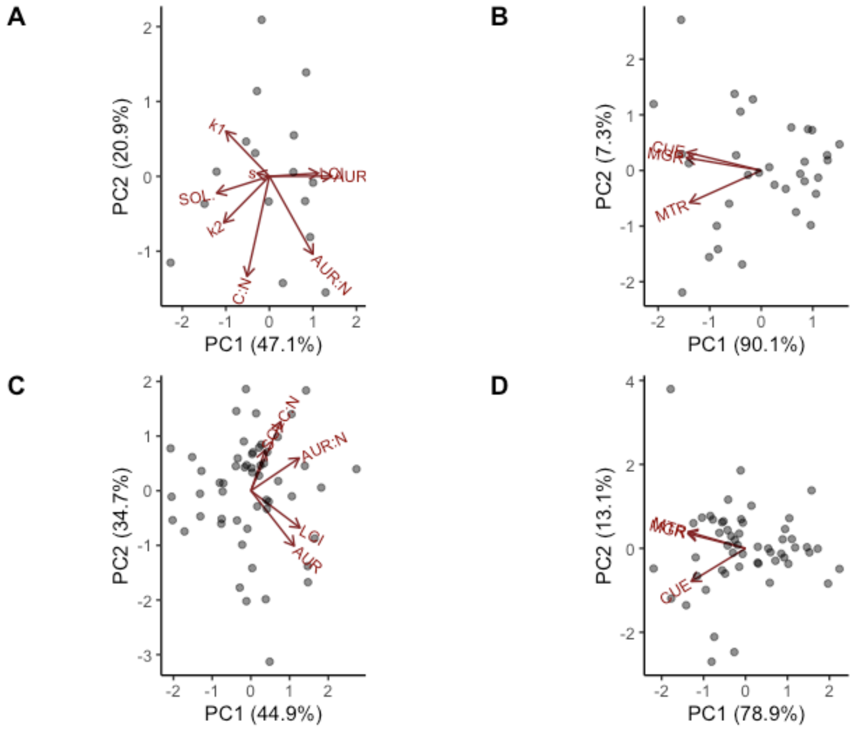

Fig. S1 Principal components analyses explaining variation in litter chemistry (C:N, AUR, AUR:N, LCI, and % soluble content [SOL.]) and decomposition parameters (k_1, k_2, and s) (A,C) and microbial physiological traits (B,D) for the microcosm experiment (A,B) and field study (C,D). See Table S1 for abbreviations.

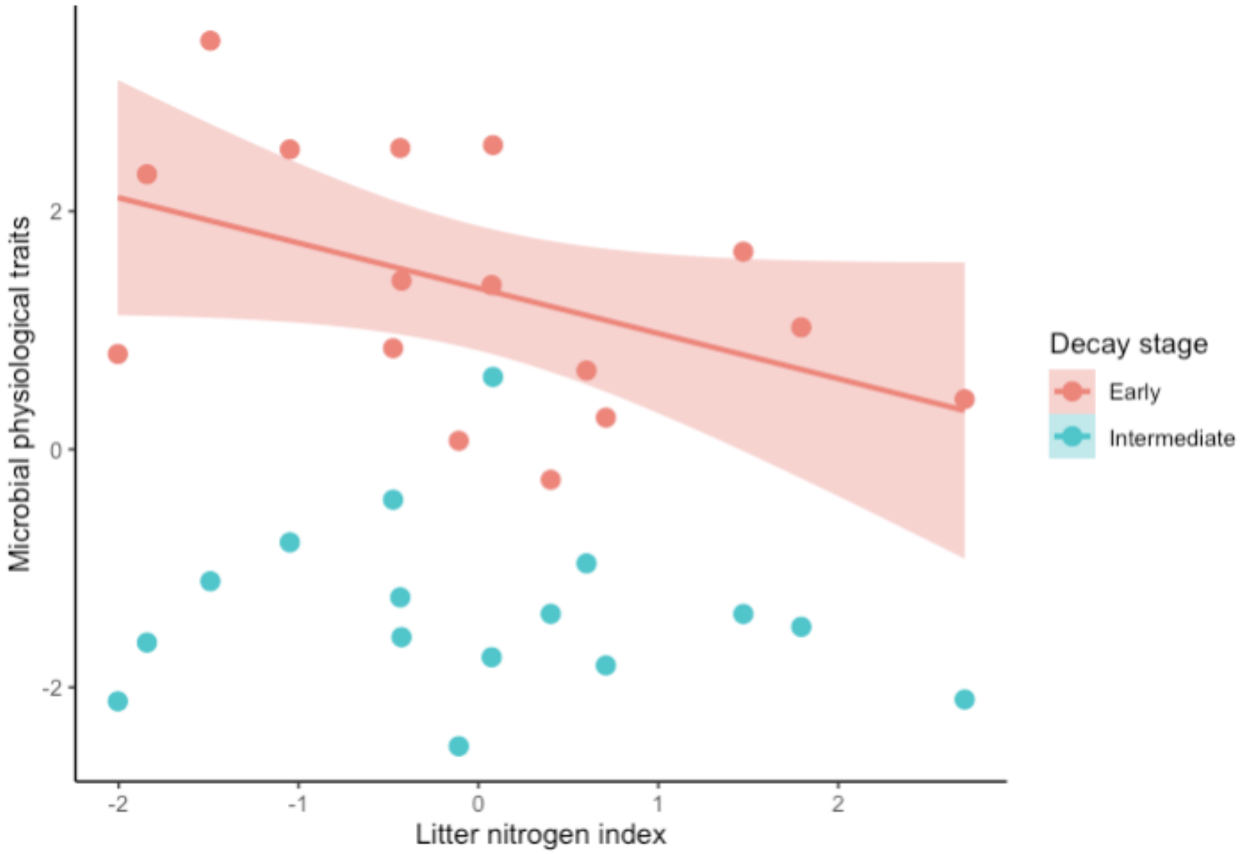

Fig. S2 Linear relationship (± SE; *n* = 16) between the litter nitrogen index (PC2 in Fig. S1A) and the microbial physiological trait index after 15 days (Early: R^2 = 0.21, P = 0.07) and 100 days (Intermediate: R^2 = 0.01, P = 0.70) of decomposition.

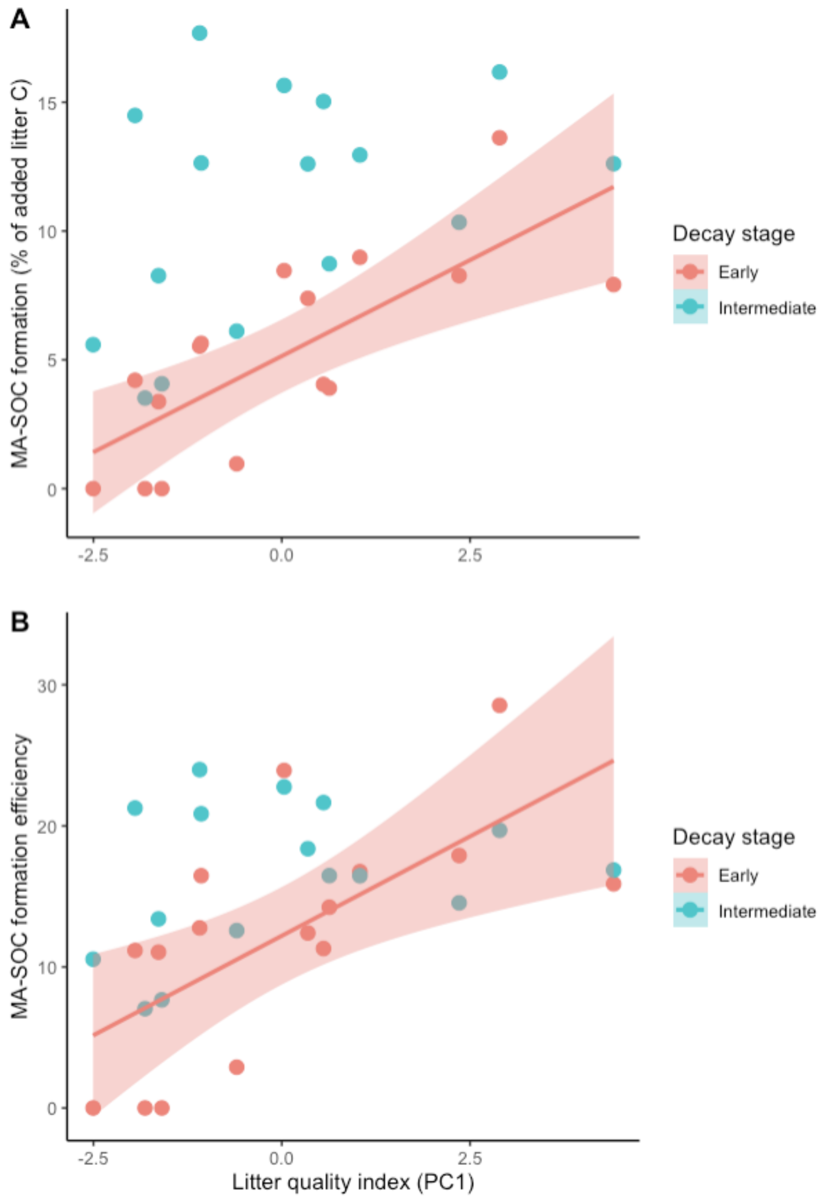

Fig S3. Linear relationship (± SE; n = 16) of the litter quality index with litter-derived mineral-associated SOC (MA-SOC) after 30 days (Early: $R^2 = 0.56$, $P < 0.01$) and 185 days (Intermediate: $R^2 = 0.17$, $P = 0.11$; A) and with mineral-associated SOC formation efficiency during early- ($R^2 = 0.44$, $P = 0.01$) and intermediate-stage decay ($R^2 = 0.07$, $P = 0.31$).

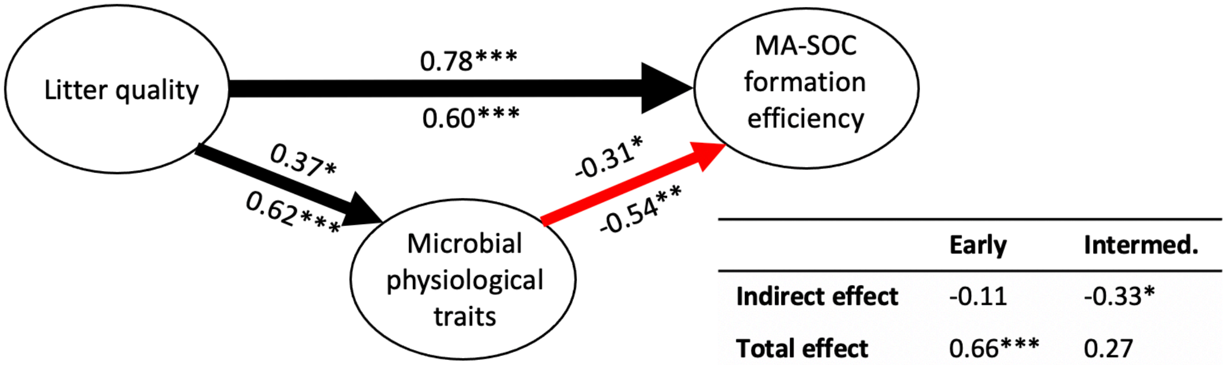

	Early	Intermed.
**Indirect effect**	-0.11	-0.33*
**Total effect**	0.66***	0.27

Fig. S4 Path analysis showing the direct and indirect effects of the litter quality index (Litter quality) on the efficiency of mineral-associated SOC formation (MA-SOC formation efficiency). Indirect effects of litter quality are mediated through the microbial physiological trait index. Numbers above and below paths represent standardized coefficients during early- and intermediate-stage decomposition, respectively, with significance levels indicated (*$p < 0.1$, **$p < 0.05$, and ***$p < 0.01$). The early and intermediate incubations were harvested on days 30 and 185, respectively, for soil C and microbial physiological traits were measured on days 15 and 100 (i.e. the approximate midpoints). Thickness and color of lines correspond to coefficient magnitude and direction, respectively. Total and indirect effects of litter quality on soil C formation are also summarized with standardized coefficients.

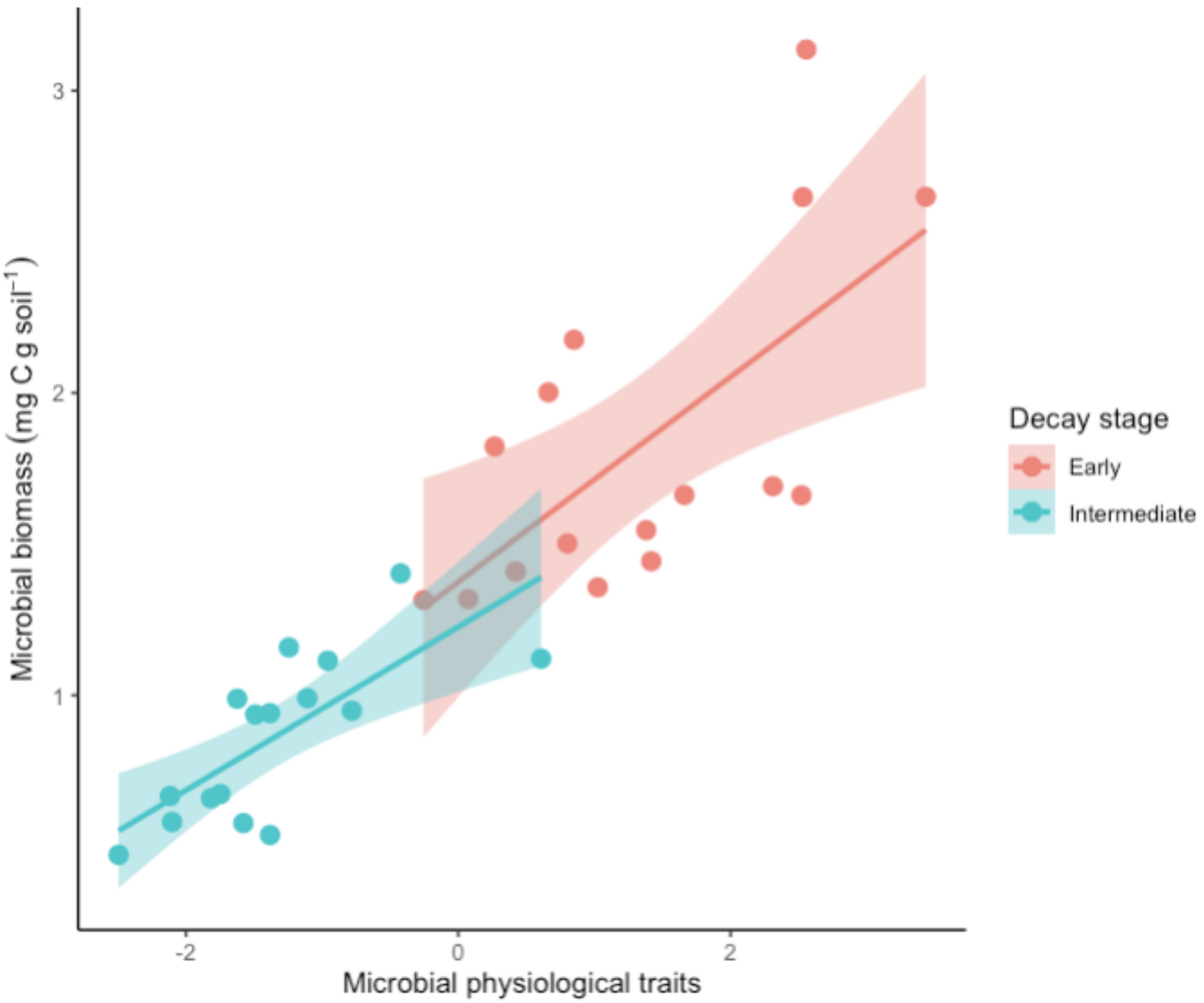

Fig. S5 Linear relationship (± SE; *n* = 16) between the microbial physiological trait index and microbial biomass after 15 days (Early: R^2 = 0.43, P = 0.01) and 185 days (Intermediate: R^2 = 0.55, P < 0.01).

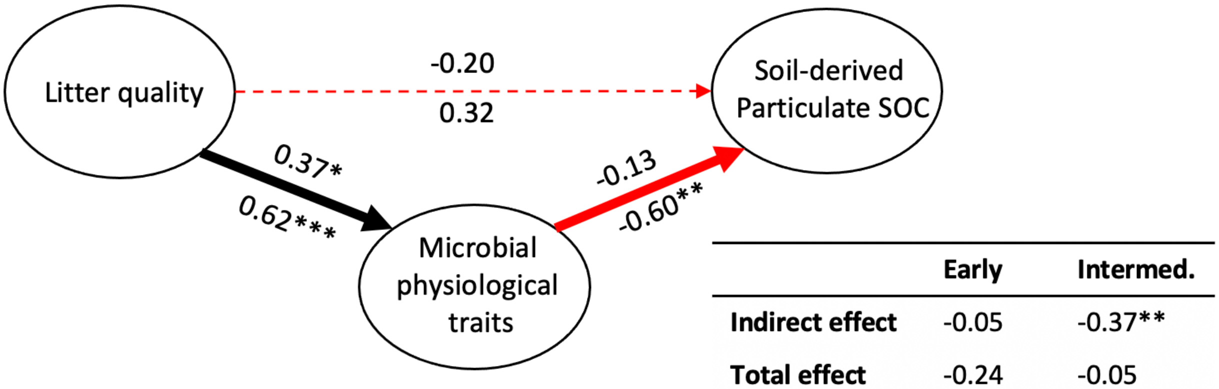

	Early	Intermed.
**Indirect effect**	-0.05	-0.37**
**Total effect**	-0.24	-0.05

Fig. S6 Path analysis showing the direct and indirect effects of the litter quality index (Litter quality) on soil-derived (i.e. pre-existing) particulate SOC (Soil-derived Particulate SOC). Indirect effects of litter quality are mediated through the microbial physiological trait index. Numbers above and below paths represent standardized coefficients during early- and intermediate-stage decomposition, respectively, with significance levels indicated (*$p < 0.1$, **$p < 0.05$, and ***$p < 0.01$). Thickness and color of lines correspond to coefficient magnitude and direction, respectively. Total and indirect effects of litter quality on soil C formation are also summarized with standardized coefficients.

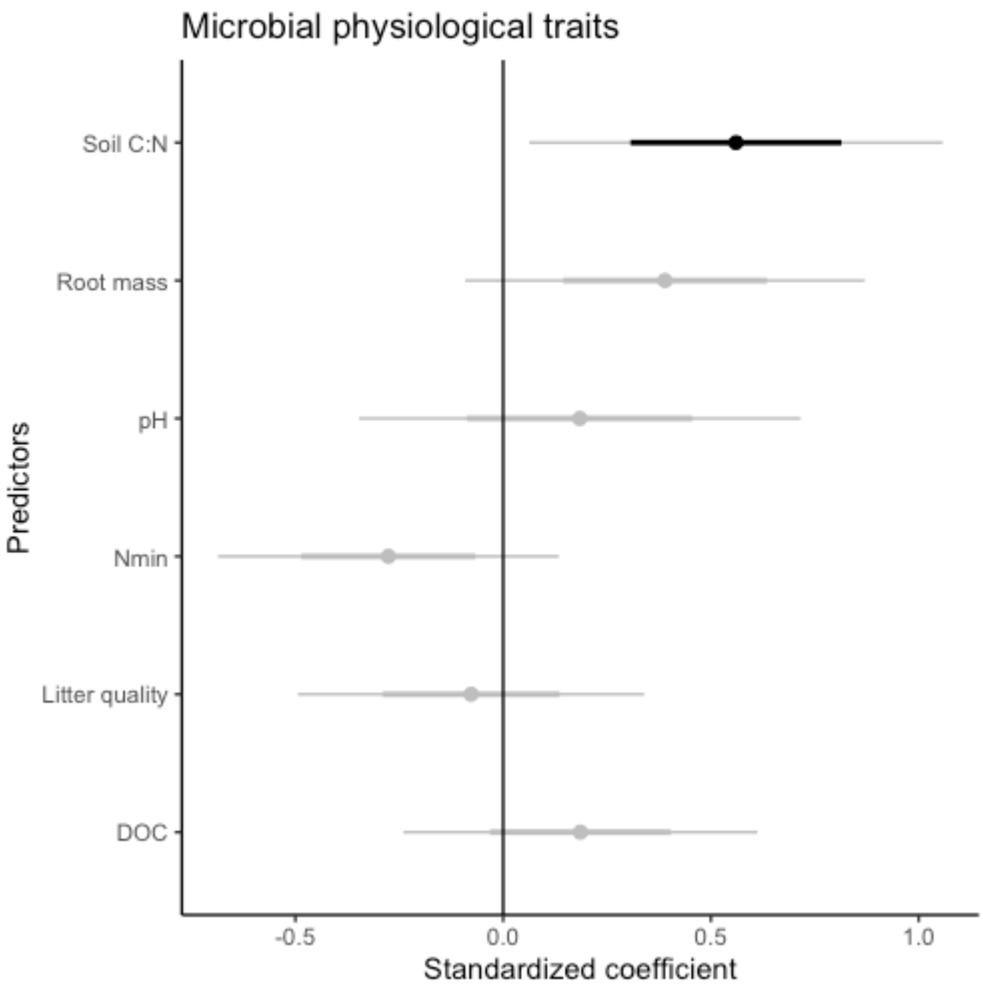

Fig. S7 Linear mixed model coefficients relating the microbial physiological trait index to soil C:N, fine root biomass (Root mass), soil pH, potential net nitrogen mineralization (Nmin), the litter quality index (Litter quality), and extractable dissolved organic carbon (DOC). Plot shows standard error (n = 54; inner bold lines) and 95 % confidence intervals (outer lines). Coefficients were centered and standardized to show the relative importance of each predictor despite the different scales on which the variables were measured.

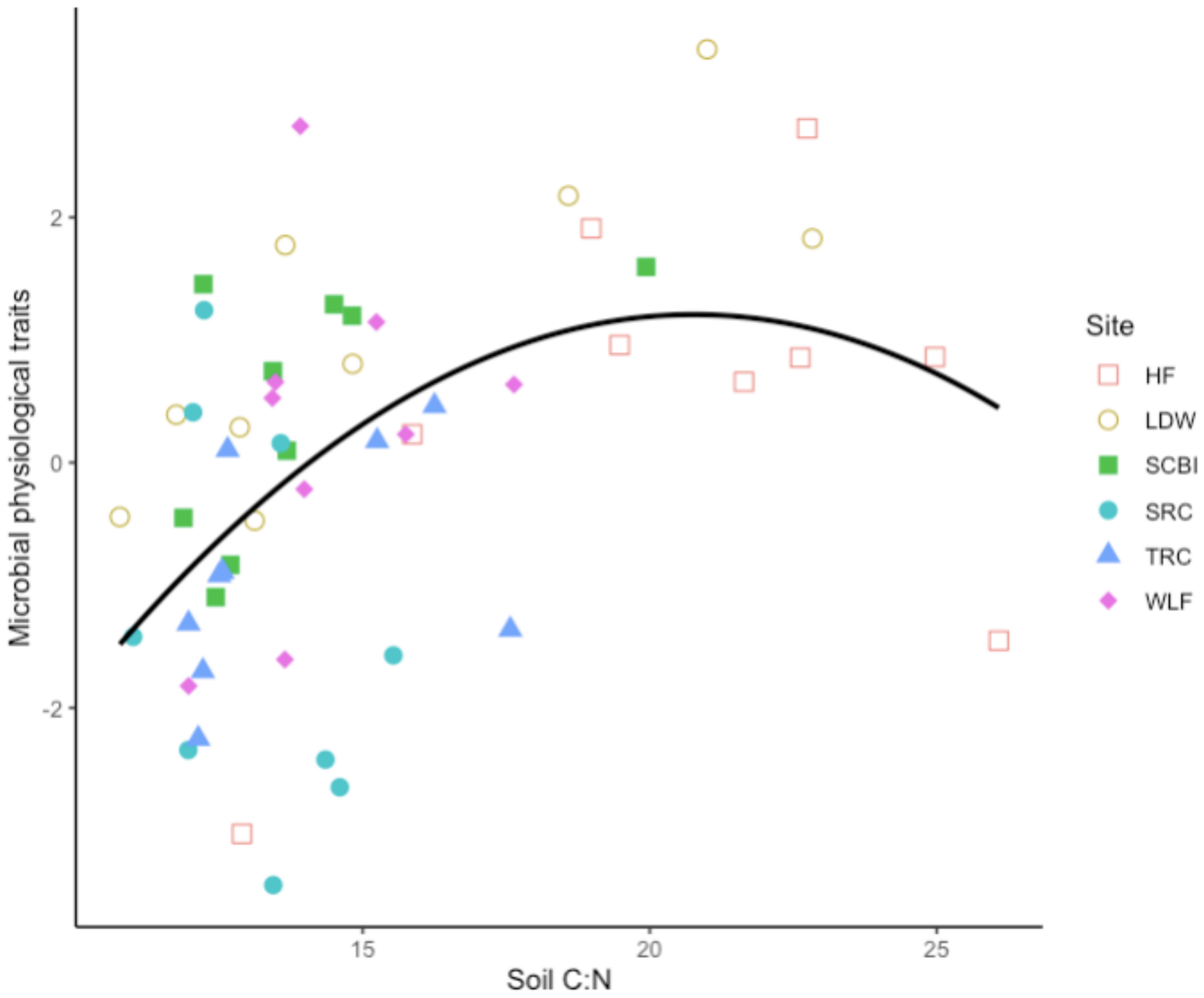

Fig. S8 Bivariate relationship between soil C:N and the soil microbial physiological trait index across 54 plots and six forests. Data are fit with a second-order polynomial (Adjusted $R^2 = 0.21$; $P < 0.01$). Site abbreviations are defined in Table S3.

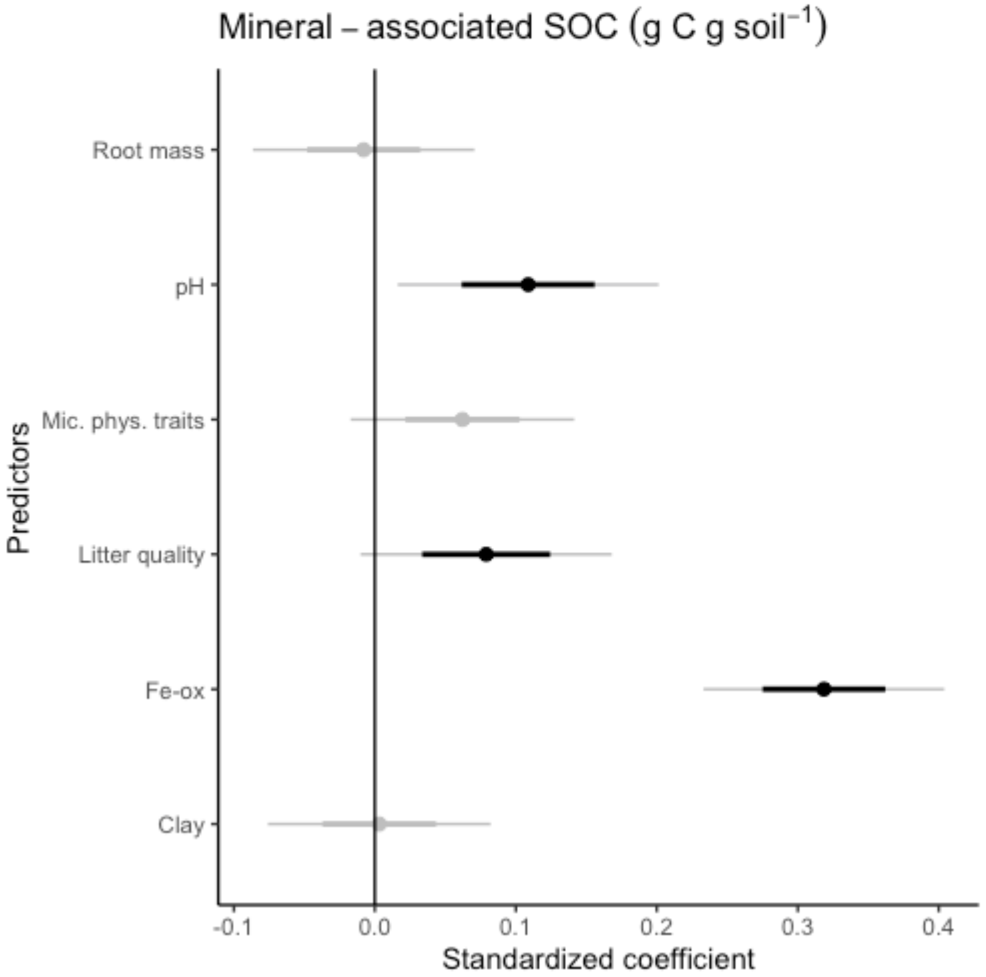

Fig. S9 Linear mixed model coefficients relating mineral-associated soil organic matter (on a per unit soil mass basis) to the litter quality index (Litter quality; PC1 in Fig. S1C) the microbial physiological trait index (Mic. phys. traits; PC1 in Fig S1D), fine root biomass, soil pH, oxalate-extractable iron (Fe-ox), and soil clay content. Plot shows standard error (n = 54; inner bold lines) and 95 % confidence intervals (outer lines).

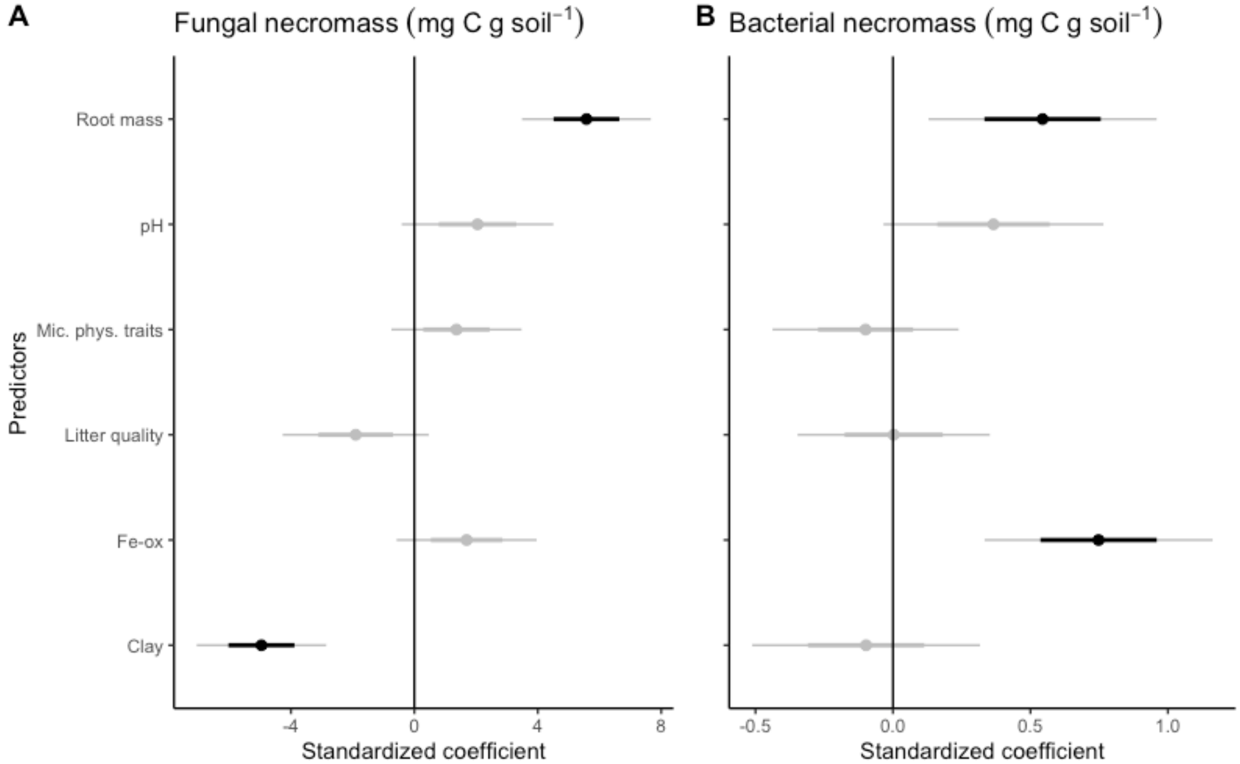

Fig. S10 Linear mixed model coefficients relating fungal necromass (A) and bacterial necromass (B) to the litter quality index (Litter quality; PC1 in Fig. S1C) the microbial physiological trait index (Mic. phys. traits; PC1 in Fig S1D), fine root biomass, soil pH, oxalate-extractable iron (Fe-ox), and soil clay content. Plot shows standard error (*n* = 54; inner bold lines) and 95 % confidence intervals (outer lines).
